# An algorithm to identify patients with treated type 2 diabetes using medico-administrative data

**DOI:** 10.1186/1472-6947-11-23

**Published:** 2011-04-14

**Authors:** Laurence M Renard, Valery Bocquet, Gwenaelle Vidal-Trecan, Marie-Lise Lair, Sophie Couffignal, Claudine Blum-Boisgard

**Affiliations:** 1 Centre for Health Studies, Public Research Centre for Health, Luxembourg; 2 EA 4069 - Epidemiology, Assessment and Health Policies, University Paris-Descartes, Paris, France; 3 Public Health Department, Faculty of Medicine, University Paris Descartes, Paris, France; 4 Risk management and quality unit, Cochin-Saint Vincent de Paul Hospital, AP-HP, Paris, France

**Keywords:** algorithm, medico-administrative data, type 2 diabetes, Europe, prevalence

## Abstract

**Background:**

National authorities have to follow the evolution of diabetes to implement public health policies. An algorithm was developed to identify patients with treated type 2 diabetes and estimate its annual prevalence in Luxembourg using health insurance claims when no diagnosis code is available.

**Methods:**

The DIABECOLUX algorithm was based on patients' age as well as type and number of hypoglycemic agents reimbursed between 1995 and 2006. Algorithm validation was performed using the results of a national study based on medical data. Sensitivity, specificity and predictive values were estimated.

**Results:**

The sensitivity of the DIABECOLUX algorithm was found superior to 98.2%. Between 2000 and 2006, 22,178 patients were treated for diabetes in Luxembourg, among whom 21,068 for type 2 diabetes (95%). The prevalence was estimated at 3.79% in 2006 and followed an increasing linear trend during the period. In 2005, the prevalence was low for young age classes and increased rapidly from 40 to 70 for male and 80 for female, reaching a peak of, respectively 17.0% and 14.3% before decreasing.

**Conclusions:**

The DIABECOLUX algorithm is relevant to identify treated type 2 diabetes patients. It is reproducible and should be transferable to every country using medico-administrative databases not including diagnosis codes. Although undiagnosed patients and others with lifestyle recommendations only were not considered in this study, this algorithm is a cheap and easy-to-use tool to inform health authorities. Further studies will use this tool with the aim of improving the quality of health care dedicated to diabetic patients in Luxembourg.

## Background

Diabetes Mellitus is a chronic disease leading to life-threatening complications.

Diabetic patients aged under 55 stratified by age and sex have a median life expectancy of 7 years less than that of non-diabetic ones [1]. The estimated prevalence for all-age-group worldwide diabetic patients, given by the World Health Organization is of 2.8% (171 million patients) in 2000 and should grow to 4.4% (366 million) in 2030 [2]. Follows the necessity to monitor the evolution of this disease and the healthcare resources dedicated to it in order to implement relevant health policies.

Type 1 and type 2 diabetes have different etiologies and physiopathologies [3] requiring different treatment, follow-up and prevention practices. Therefore, in order to better inform health authorities to implement relevant health policies, type 1 and type 2 diabetes have to be distinguished, as suggested by Guttman *et al. *[4]. Data used to determine these policies are either based on clinical information or on medico-administrative data. Clinical data is collected on population samples carrying risks of selection bias, using patients' records, biological samples or self-administered questionnaires. This data collection is time-consuming and expensive, and does not guarantee accurate and exhaustive datasets. Despite their limits, several studies have found that administrative datasets were a useful source of information for diabetes surveillance [4-6]. The distinction between the two types of diabetes can be achieved either by a physician's diagnosis or by an epidemiological algorithm [7].

The literature provides some epidemiological algorithms based on administrative data [5,7,8]. Authors used either age at diagnosis or diagnosis codes of diabetes. However, medico-administrative data does not always include such medical information. This is the case in some European countries, among them France and Luxembourg. Luxembourg national authorities are currently developing projects to implement public health policies directed toward type 2 diabetes but no accurate prevalence rates are repeatedly available for international comparisons [9].

The objectives of this study were to develop a validated algorithm in order to detect treated diabetic patients using health insurance claims and identify patients with type 2 diabetes as well as to estimate the annual prevalence of treated type 2 diabetes in Luxembourg.

## Methods

### Design of the study

A 3-step epidemiological algorithm called DIABECOLUX algorithm was developed to identify patients with treated diabetes and to distinguish type 2 diabetic patients, using administrative reimbursement databases. It was applied to the population residing in the Grand Duchy of Luxembourg covered by the national health insurance over the period 2000-2006.

### Setting and data sources

The national health insurance of Luxembourg is a compulsory regime, covering 95% of the resident population. Its claim database is representative in terms of age and sex of the whole population of Luxembourg [10]. For practical reasons (storage space, processing time), a preliminary step selected from the total population (N = 484,560) the exhaustive population, who has been reimbursed at least one hypoglycemic agent (Anatomical Therapeutic Chemical (ATC) classification: A10) [11], either per os or injectable (i.e. insulin), over 2000-2006 (N = 28,269). This preliminary selection, based on the hypothesis that a treated diabetic patient had at least one hypoglycemic agent reimbursed over the period, included all the treated diabetic patients and allowed more rapidity in the execution of the algorithm. Information about reimbursed medical acts, consultations, treatments, hospitalizations, biological analyses was provided for that period by the Inspection Générale de la Sécurité Sociale (Ministry of the national health insurance). Moreover, information about hypoglycemic treatment during the period 1995-1999 was added to the dataset for the selected population in order to check the history of its diabetic status. Then datasets were crossed with death certificate databases to check patients' vital status. Since all patients were given a 22-digit identification number to ensure secured anonymization and that the identity of the patients could not be retrieved by database crossing, no ethical or data protection approval was required.

### Definition of treated diabetes

The definition of treated diabetic patients that was considered in this study finds its roots in the methodology of the ENTRED study [12-14], a validated transversal epidemiological study defining treated diabetic patients as having at least three A10 reimbursements in the year, to avoid false positive patients (prescription and diagnosis errors). Since the DIABECOLUX study was longitudinal, the above definition was softened to take into account the continuity of the treatment. Therefore, a treated diabetic patient was defined as fulfilled at least one of the following four criteria:

▪ Criterion 1: 3 deliveries (or more) of A10 per year, for 2 years or more;

▪ Criterion 2: 3 deliveries of A10 (or more) for 1 year AND 2 deliveries per year, for 2 years or more;

▪ Criterion 3: 2 deliveries of A10 per year, for 3 years or more - to consider patients often abroad;

▪ Criterion 4: 3 deliveries of A10 (or more) for the year of Death (X), OR the year X-1, OR 2005, OR 2006 - to consider the right truncation and include the incident cases of the last years of the period.

### Algorithm steps

The first step of the algorithm was divided into two phases. Its aim was to identify the patients treated for either type 1 or 2 diabetes between 2000 and 2006 in a first phase and 1995-2006 in a second phase for patients dead in the early 2000's and not included in the first phase.

The second step of the algorithm selected within the diabetic population patients who received oral hypoglycemic agents (OHAs, ATC classification: A10B) over 1995-2006. The remaining patients only received insulin (A10A) over that period.

To determine a threshold dividing type 1 and type 2 diabetic patients among the latter, a subpopulation (N = 229) treated with solely insulin after having received OHAs over 1995-2006 was selected. The mean age (y) of this subpopulation, on the year of treatment change, was calculated at 66.3. The third step included patients older than 66, the year of their first insulin delivery between 1995 and 2006.

Patients included in the second and the third steps constituted the DIABECOLUX population, considered treated for type 2 diabetes in Luxembourg over 2000-2006.

### Algorithm validity

The relevance and accuracy were checked for each step of the algorithm. The sensitivity (SE), the specificity (SPE) and the positive and negative predictive values (PPV and NPV) of the process of distinction between type 1 and type 2 diabetes were estimated. Since no gold standard was available, the range of the possible values of the real proportion of type 2 diabetic patients (T2P) was estimated in the population treated for diabetes in Luxembourg. This range was based on the characteristics of treated diabetic patients i.e. the estimated proportion of type 2 diabetic patients by age group, the mean age of patients at the time of transition from OHA to solely insulin treatment (mean age = 66) and the minimum age at the time of this transition (age = 27). Since T2P was not homogeneous over the ages of the diabetic population, it was decomposed for age classes [0-27[; [27-66[ and [66-100]. A range of possible values for the decomposed T2P was estimated. Ranges of possible true and false positives and negatives were therefore estimated for each age class based on the relative ranges of decomposed T2P and the extreme values found in the algorithm (i.e. everybody wrongly or properly included), and then pooled to calculate the intervals of SE, SPE, PPV and NPV. This process was applied to various possible values of the global T2P. The algorithm remained constant during this process.

### Prevalence estimation

Patients were defined as having treated type 2 diabetes each year since their first A10 delivery until the end of the period or their death. Treated type 2 diabetes prevalence for year X was defined as the number of patients treated for type 2 diabetes in X divided by the resident population covered by the national health insurance on 31^st ^December of that year. Prevalence rates were estimated for each year from 2000 to 2006. Prevalence rates were not estimated for the 1995-1999 period, since data was only available for patients treated between 2000 and 2006.

Intra-country validation was performed using as a comparator the prevalence estimated in the ORISCAV-LUX study [15,16]. This validated study is an observational cross-sectional study collecting data from biological samples and self-administered questionnaires of a sample of 1,432 subjects aged from 18 to 69. The ORISCAV-LUX population was representative of the residing population in Luxembourg (2001 census) in terms of sex, age and residence district. Since prevalence was estimated for both type 1 and type 2 diabetes, type 2 diabetes prevalence was calculated using the proportion of type 2 diabetic patients among the ENTRED population, set at 92% [8]. To be comparable with ORISCAV-LUX, DIABECOLUX prevalence was re-estimated on the population aged from 18 to 69 years and projected in 2008 from 2000-2006 data. The projection method was a double exponential smoothing (with the lowest Root Mean Square Error to minimize prediction errors) since the observed trend was linear (R^2 ^= 0.96).

Finally, the prevalence by age class was estimated for males and females for year 2005.

### European comparisons

To compare the annual prevalence of type 2 diabetes in Luxembourg with that of neighboring countries, a direct age-standardized rate from the European Union 15-country (EU15) population [17] was applied to Luxembourg, Belgium [18] and France [19-21]. 2000-Eurostat prevalence rates [22] had already been age-standardized using the same method.

When both type 1 and 2 diabetes were considered in European prevalence rates, the percentage of 92% was applied as for ORISCAV-LUX prevalence.

### Statistical procedures

A specific missing data analysis was performed to analyze data quality.

Standard descriptive statistics were used to analyze the database. When appropriate, a 95% confidence interval [95% CI] was provided.

Population selection, data cleaning and statistical analyses were performed using SAS^® ^9.2. package.

## Results

**Figure 1:**
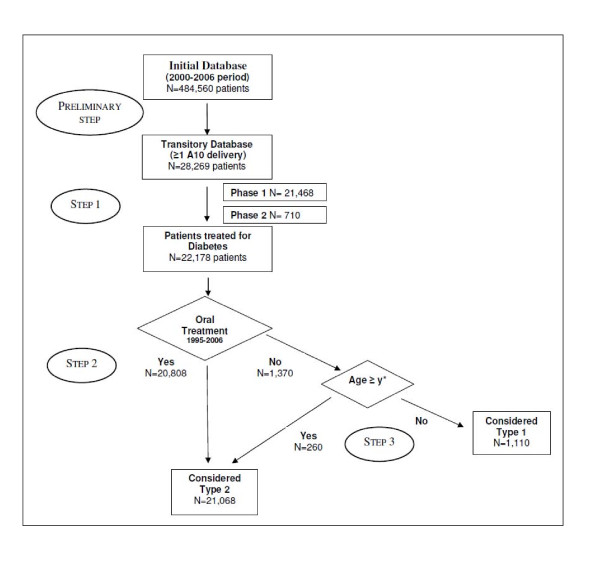
**The DIABECOLUX algorithm applied to Luxembourg**. * with y determined by the local physicians' practices and estimated at 66.3 years old in Luxembourg.

Figure 1 shows the DIABECOLUX algorithm applied to the population of Luxembourg. Step 1 included all the patients treated for diabetes in Luxembourg (N = 22,178). The first phase of step 1 included 96.8% (N = 21,468) of this population. Among those, 83.4% met criterion 1, 7.8% for criterion 4, 5.3% for criterion 2 and 0.3% for criterion 3. Around 3% (N = 710) were added in the second phase of step 1. Among the 22,178 patients treated for diabetes, step 2 selected 20,808 patients and step3 added 260 patients.

Consequently, the DIABECOLUX database was constituted by 21,068 patients (95% of the total population treated for diabetes) residing in Luxembourg and defined as treated for type 2 diabetes, for the 2000-2006 period. The number of patients in the initial exhaustive population, in the population treated for diabetes and in the population treated for type 2 diabetes, over the 2000-2006 period and for each year is presented in Table 1.

**Table 1:** Number of patients in the initial exhaustive population, in the population treated for diabetes and in the population treated for type 2 diabetes in Luxembourg, over the 2000-2006 period and for each year.

		2000-2006	2000	2001	2002	2003	2004	2005	2006
**Initial Population**		484,560	418,182	424,037	428,457	433,424	439,628	444,783	450,000
**Treated Diabetes**		22,178	13,416	14,277	14,956	15,489	16,241	17,172	17,522
**Treated Type 2 Diabetes**		21,068	13,152	13,944	14,704	15,269	16,026	16,751	17,070

### Validation of the DIABECOLUX algorithm

The missing data analysis revealed 100% of data available for all the variables used in the algorithm.

Among the 6,091 patients rejected in step 1, 92 (0.04%) had a hospital discharge of Diabetes Mellitus (ICD10 classification: E10-E14) [21] during the period.

The proportion T2P was estimated between 92.8% and 96.7%. Varying T2P values, SE was always estimated higher than 98.2%, SPE always higher than 63.5%, PPV always higher than 97.7% and NPV always higher than 65.9% (Table 2).

**Table 2:** Sensitivity (%) and Specificity (%) of the DIABECOLUX algorithm for various possible proportions (T2P) of type 2 diabetes in the diabetic population (%).

**T2P**^**†**^	**TP**^**†**^	**TN**^**†**^	T2P.POPtot	(1-T2P).POPtot	SE = TP/(T2P.POPtot)	SPE = TN/((1-T2P).POPtot)	PPV = TP/Pos	NPV = TN/Neg
Min: 92.8%	20,581	1,110	20,581	1,597	100	69.5	97.7	100
93%	[20,581-20,626]	[1,067-1,110]	20,626	1,552	[99.8-100]	[68.7-71.5]	[97.7-97.9]	[96.1-100]
94%	[20,581-20,847]	[845-1,110]	20,847	1,331	[98.7-100]	[63.5-83.4]	[97.7-99.0]	[76.1-100]
95%	[21,036-21,068]	[731-1,109]	21,069	1,109	[99.8-100]	[65.9-100]	[99.8-100]	[65.9-99.9]
96%	[21,063-21,068]	[731-887]	21,291	887	[98.9-99.0]	[82.4-100]	100	[65.9-79.9]
Max: 96.7%	21,068	[731-732]	21,446	732	98.2	[99.9-100]	100	65.9

**T2P**: Proportion of type 2 diabetes in the treated diabetic population; **TP**: True Positive; **TN**: True Negative; **POPtot**: Total diabetic population (POPtot = 22,178); **SE**: Sensitivity; **SPE**: Specificity; **PPV**: Positive Predictive Value; **Pos**: Population included (Pos = 21,068); **NPV**: Negative Predictive Value; **Neg**: Population excluded (Neg = 1,110).

^**† **^: pooled from age classes.

For the intra-country validation, the prevalence projection of treated type 2 diabetes for 18 to 69 year-old patients was estimated in 2008 at 3.5% [95% CI: 3.4-3.5] vs. ORISCAV-LUX: 2.6% [95% CI: 2.0- 3.5]; for males 4.1% [95% CI: 4.1-4.2] vs. 3.2% [95% CI: 2.2-4.7]; and for females 2.8% [95% CI: 2.7-2.8] vs. 2.0% [95% CI: 1.3-3.1].

### Prevalence of treated type 2 diabetes

The prevalence of treated type 2 diabetes in Luxembourg was estimated for the DIABECOLUX population at 3.15% (N = 13,152) in 2000 increasing linearly to 3.79% (N = 17,070) in 2006, with a mean annual increase of 3.2%. The age of the population ranged between 12 and 100 (median age: 66) in 2000, remaining constant over the period. There were 50.6% of males in 2000 and 53.5% in 2006.

The EU15 age-standardized Luxembourg prevalence rates were estimated at 4.20% in 2004 and 4.64% [95% CI: 4.52-4.77] in 2007, which was not significantly different from Belgium (3.6% [95% CI: 2.7-5.2]) in 2004 and slightly higher than France (4.50%) in 2007.

**Figure 2:**
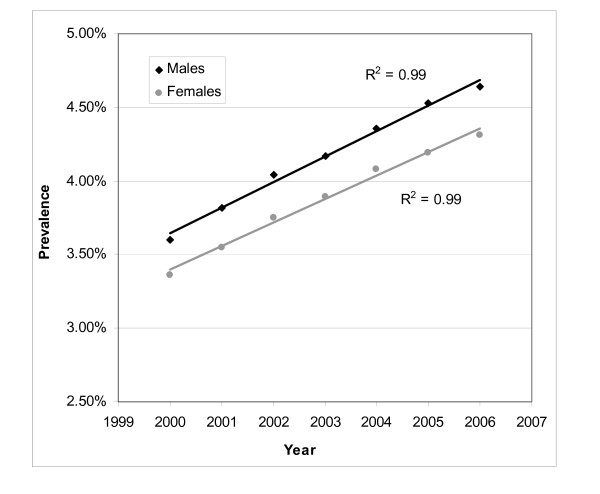
**Evolution of the prevalence of treated type 2 diabetes in Luxembourg by sex over the 2000-2006 period (N = 21,068), age-standardized over the EU15 population**.

Male and female prevalence rates, age-standardized over the EU15 population, had a linear (R^2 ^= 0.99 for both) and increasing trend (Figure 2) over time. Prevalence rates were respectively 3.6% and 3.4% in 2000 and increased during the period to reach respectively 4.6% and 4.3% in 2006. The mean annual increase in the prevalence between 2000 and 2006 was estimated at 4.3% for males and 4.2% for females (respectively 4.7% and 4.5% for the 2000-2005 period). For the 2000-2005 period, the mean annual increase in the EU15-age standardized French prevalence was estimated at respectively 6.1% and 5.7%.

French and Luxembourg prevalence rates were found significantly different for males and for females (p < 0.05), Luxembourg prevalence rates being higher in both cases. Moreover, Luxembourg female and French male prevalence rates were found not significantly different.

**Figure 3:**
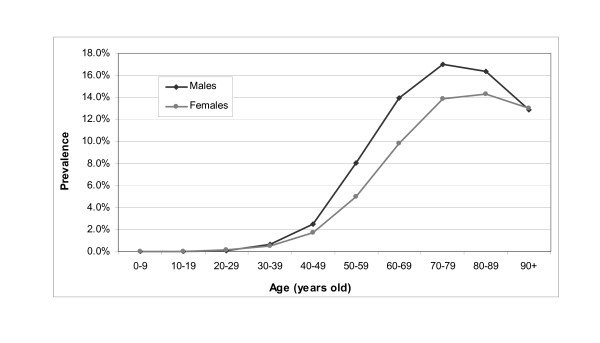
**Prevalence of treated type 2 diabetes in Luxembourg by age class and sex in 2005 (N = 16,288)**.

Prevalence rates according to ages were similar for both males and females in 2005 (Figure 3). They were low and stable until 40. From 40 to 80, prevalence rates increased rapidly, with an increasing gap between males and females. This gap reached a maximum of 4.1 points for the 60-69 age class. An inflection was initiated in both curves in the 60-69 age class, leading to a decrease after a peak at 70 years old for males (17.0%) and 80 years old for females (14.3%).

## Discussion

The application of the algorithm selected 21,068 patients treated for type 2 diabetes over the 2000-2006 period in the population of Luxembourg. Prevalence rates estimated from DIABECOLUX and ORISCAV-LUX [15,16] were found not significantly different, thereby validating the algorithm for the relative population. Aiming at improving knowledge to implement and assess health policies, this algorithm required both good sensitivity and a positive predictive value to identify type 2 diabetic patients among treated diabetic population. They were found respectively superior to 98.2% and 97.7% with a specificity superior to 63.5%. To our knowledge, there is no study about the sensitivity and the specificity concerning similar algorithms in the literature.

It also appeared difficult to find comparable European prevalence rates in the literature. Most studies did not provide EU15 age-standardized data or enough details to standardize data. Unfortunately, studies with age-standardized prevalence rates used different methodologies in terms of population samples or data collection [9,23-25]. Therefore, the final choice regarding European comparisons was directed towards official data.

The EU15-age-standardized prevalence in Luxembourg was 3.5% in 2000, close to that in its neighboring countries (France: 3.0%, Belgium: 3.2%, Netherlands: 3.5%, Germany: 4.0%) [22] and not significantly different from Belgium in 2004. French and projected Luxembourg prevalence rates in 2007 were found to be very close.

Since the prevalence rates provided in the literature involved both type 1 and 2 diabetes, the proportion of type 2 in the diabetic population, which stands at 92% as estimated by ENTRED, had to be applied. This percentage was estimated at 90% by WHO [26] and 95% by the Ng-Dasgupta-Jonhson algorithm [7]. The DIABECOLUX algorithm estimated it at 95%. Applying 95% to prevalence rates did not change the results of comparisons.

As a comparison, both French [20] and Luxembourg prevalence rates (EU15 age-standardized) increased linearly over 2000-2005 with a greater mean annual increase in prevalence for France for both genders. This can be the result of the screening campaigns implemented in France during this period. Moreover, the Luxembourg prevalence was found significantly higher than the French one for both genders. However, this gap is less obvious for regions bordering Luxembourg [27,28]. After comparison of prevalence rates for France and Luxembourg in 2005 by age and sex, no significant difference was found for male until 80 and female until 60. Over 80, French prevalence rates decreased more rapidly, which could be explained by a greater incidence of treated patients in Luxembourg.

Since no published data on diabetes is available for Luxembourg [9], this tool can generate some and can help policy-makers to follow the trend of this pathology.

Focusing on the database and algorithm limitations, only diagnosed patients who are treated and reimbursed were considered in this study. Undiagnosed patients and those only under lifestyle recommendations were estimated at 1.5% of the 18-69 year-old diabetic patients in Luxembourg in 2008 [16]. Drugs brought abroad cannot be identified in the database used. However, the induced underestimation of the prevalence is negligible (high reimbursement rate and residing population). Using longitudinal data allowed avoiding prescription errors. Finally, declaration for drug reimbursement being automatically done by pharmacists limited the risk of errors.

The definition of a diabetic patient was based on the number of A10 deliveries, since the diagnosis code was not available in the claim database of Luxembourg and hospital discharge codes were not always valid or accurate. As in Guttmann *et al. *study [4], where the biggest sum of sensitivity and specificity was for 3 claims, it was set at 3 deliveries per year to limit prescription errors. This restriction was relaxed to 2 deliveries for at least 3 years to include subjects who often travel abroad. Finally, the algorithm misclassifies all type 1 diabetic patients over 66, by wrongly categorizing a maximum of 260 patients under the 'type 2 diabetes' category. However, since the proportion of type 1 is low and the complications and prevention policies are similar in this age class, the impact of this misclassification is limited.

The DIABECOLUX algorithm was based on deterministic conditions leading to the reproducibility of the results and the possibility to be applied every year. Since the mean age (y) of the solely insulin treatment start (i.e. 66 in the population of Luxembourg) was based on national medical practices and not determined by a fixed threshold, it allows the transferability of the DIABECOLUX algorithm to other countries.

## Conclusion

The DIABECOLUX algorithm was developed to distinguish type 2 diabetes patients using medico-administrative data. Validated by medical data in Luxembourg, it appears to be a good tool to estimate the prevalence of treated diabetes type 2 with a sensitivity and a positive predictive value respectively greater than 98.2% and 97.7%. This algorithm is reproducible and should be transferable to every country using medico-administrative data, presenting an advantage in terms of costs of data collection. It is a useful tool for health authorities to follow the evolution of type 2 diabetes and evaluate health policies.

Further studies will focus on the quality of health cares dedicated to type 2 diabetic patients in Luxembourg with regard to European guidelines.

## Abbreviations

ATC classification: Anatomical Therapeutic Chemical classification; CI: Confidence Interval; CNAMTS: Caisse Nationale de l'Assurance Maladie des Travailleurs Salariés (French National Health Insurance Fund for employees); EU15: European Union 15-country population; ICD10 classification: International Classification of Diseases 10^th ^revision; NPV: Negative Predictive Value; OHA: Oral Hypoglycemic Agent; PPV: Positive Predictive Value; RSI: Régime Social des Indépendants (French Self-employed Health Insurance); SE: Sensitivity; SPE: Specificity; T2P: Proportion of Type 2 diabetic patients; WHO: World Health Organization

## Competing interests

The authors declare that they have no competing interests.

## Authors' contributions

LMR, VB, GVT wrote the manuscript. LMR, MLL, GVT, CBB designed and collected data. VB performed the statistical analyses. SC contributed to the methodology design. CBB, MLL, SC reviewed/edited the manuscript. All authors read and approved the final manuscript.

## Pre-publication history

The pre-publication history for this paper can be accessed here:

http://www.biomedcentral.com/1472-6947/11/23/prepub
